# CCNE1 is a predictive and immunotherapeutic indicator in various cancers including UCEC: a pan-cancer analysis

**DOI:** 10.1186/s41065-023-00273-0

**Published:** 2023-03-24

**Authors:** Xingyu Zheng, Lingli Chen, Wenlu Liu, Shuangshuang Zhao, Ye Yan, Jianzhen Zhao, Wenyan Tian, Yingmei Wang

**Affiliations:** 1 Department of Gynecology and Obstetrics, Tianjin Medical University General Hospital, Tianjin, China; 2 Tianjin Key Laboratory of Female Reproductive Health and Eugenics, Tianjin Medical University General Hospital, Tianjin, China

**Keywords:** CCNE1, Pancancer, Prognosis, Immune, UCEC

## Abstract

**Background:**

CCNE1 plays an important oncogenic role in several tumors, especially high-stage serous ovarian cancer and endometrial cancer. Nevertheless, the fundamental function of CCNE1 has not been explored in multiple cancers. Therefore, bioinformatics analyses of pan-cancer datasets were carried out to explore how CCNE1 regulates tumorigenesis.

**Methods:**

A variety of online tools and cancer databases, including GEPIA2, SangerBox, LinkedOmics and cBioPortal, were applied to investigate the expression of CCNE1 across cancers. The pan-cancer datasets were used to search for links between CCNE1 expression and prognosis, DNA methylation, m6A level, genetic alterations, CCNE1-related genes, and tumor immunity. We verified that CCNE1 has biological functions in UCEC cell lines using CCK-8, EdU, and Transwell assays.

**Results:**

In patients with different tumor types, a high mRNA expression level of CCNE1 was related to a poor prognosis. Genes related to CCNE1 were connected to the cell cycle, metabolism, and DNA damage repair, according to GO and KEGG enrichment analyses. Genetic alterations of CCNE1, including duplications and deep mutations, have been observed in various cancers. Immune analysis revealed that CCNE1 had a strong correlation with TMB, MSI, neoantigen, and ICP in a variety of tumor types, and this correlation may have an impact on the sensitivity of various cancers to immunotherapy. CCK-8, EdU and Transwell assays suggested that CCNE1 knockdown can suppress UCEC cell proliferation, migration and invasion.

**Conclusion:**

Our study demonstrated that CCNE1 is upregulated in multiple cancers in the TCGA database and may be a promising predictive biomarker for the immunotherapy response in some types of cancers. Moreover, CCNE1 knockdown can suppress the proliferation, migration and invasion of UCEC cells.

**Supplementary Information:**

The online version contains supplementary material available at 10.1186/s41065-023-00273-0.

## Introduction

Cyclin E1 (CCNE1), also known as CCNE and pCCNE1, is an oncogenic driver gene that is linked to a poor prognosis and platinum resistance in certain malignancies [1, 2]. CCNE1 is amplified in 8% of endometrial endometrioid adenocarcinomas (EMCAs), 45% of uterine carcinosarcomas, and 50% of serous EMCAs, particularly in high-grade serous ovarian cancer (HGSOC) and endometrial cancer (EC) [3–6]. The protein CCNE1 forms complexes with CDK2 to facilitate the cell cycle switch from the G1 phase to the S phase [7]. CCNE1 overexpression increases stress at replication forks and increases the likelihood of double-strand breaks (DSBs), which are repaired through homologous recombination with high fidelity [8].

Numerous studies have shown that CCNE1 is significant for the occurrence and progression of various tumors [6, 9]. Nicholas C. Turner et al. found that activation of CDK2 bypass induced by high expression of CCNE1 led to palbociclib resistance in breast and ovarian cancer cell line models [7]. In addition, Khaled Aziz et al. found that more liver cells of CCNE1-overexpressing mice (vs. control mice) had polyploidy, loss or increase of entire chromosomes, oxidative stress and DNA damage, and CCNE1 facilitated development of liver tumors; these results have also been found in human HCC cells [8]. Moreover, research has shown that MYLK-AS1 silencing can inhibit CCNE1 expression via the transcription factor TCF7L2, thereby regulating the cell cycle distribution and proliferation of nephroblastoma cells [10]. At present, there are no complete bioinformatics analyses regarding the biological function and role of CCNE1 in multiple tumors.

To verify the biological functions and prognostic value of CCNE1 in numerous malignancies, systematic bioinformatics analysis was conducted in this study using patient data from numerous databases. The relationships between CCNE1 expression and prognosis, DNA methylation, genetic modifications, m^6^A analysis, CCNE1-related gene expression levels, and tumor immunity were assessed, and the functional importance of altered expression levels of CCNE1 in diverse malignancies was thoroughly examined. Through in vitro experiments, we specifically examined the connection between CCNE1 expression and the occurrence and cell proliferation of UCEC.

## Materials and methods

### Analysis of CCNE1 mRNA expression and survival prognosis

SangerBox (http://SangerBox.com/Tool) was used to explore the expression of CCNE1 between tumor tissues and normal tissues in 27 tumor patients based on The Cancer Genome Atlas (TCGA) and Genotype Tissue Expression (GTEx) datasets [11]. These 27 tumors included adrenocortical carcinoma (ACC), breast invasive carcinoma (BRCA), bladder urothelial carcinoma (BLCA), colon adenocarcinoma (COAD), cholangiocarcinoma (CHOL), cervical squamous cell carcinoma and endocervical adenocarcinoma (CESC), glioblastoma multiforme (GBM), esophageal carcinoma (ESCA), kidney chromophobe (KICH), kidney renal clear cell carcinoma (KIRC), renal papillary cell carcinoma (KIRP), head and neck cancer (HNSC), brain lower grade glioma (LGG), liver hepatocellular carcinoma (LIHC), kidney acute myeloid leukemia (LAML), lung squamous cell carcinoma (LUSC), lung adenocarcinoma (LUAD), ovarian serous cystadenocarcinoma (OV), prostate adenocarcinoma (PRAD), pancreatic adenocarcinoma (PAAD), rectum adenocarcinoma (READ), stomach adenocarcinoma (STAD), skin cutaneous melanoma (SKCM), thyroid carcinoma (THCA), testicular germ cell tumors (TGCT), uterine carcinosarcoma (UCS) and uterine corpus endometrial carcinoma (UCEC). CCNE1 expression between normal and tumor tissues or in different tumor stages was analyzed in UCEC via the TCGA database (https://xenabrowser.net/datapages/). Using the GEPIA2 portal (http://gepia2.cancer-pku.cn/#index), the association between CCNE1 expression and tumor stage was explored in various cancers, including ACC, BLCA, CHOL, HNSC, KICH, KIRC, KIRP, LIHC, LUAD, OV, READ and TGCT [12]. Through the GEPIA2 online platform, overall survival (OS) and disease-free survival (DFS) maps of CCNE1 were obtained in multiple cancers via TCGA datasets.

Furthermore, the "Gene-KM plotter" module was used for COX_OS analysis data of CCNE1 for various cancers via SangerBox. The relationship between CCNE1 expression and prognosis in ovarian cancer, breast cancer, gastric cancer, lung cancer, and liver cancer was then analyzed using the Kaplan‒Meier plotter portal (https://kmplot.com/analysis/) [13].

### DNA methylation analysis and genetic alteration analysis

We compared the DNA promoter methylation levels of CCNE1 between various cancers and normal tissues in TCGA datasets through the UALCAN portal [14]. The methylation levels of row data from TCGA were normalized using TPM in the databases. DNA methylation levels at the CCNE1 promoter were examined for thirteen tumors. In the DepMap portal (https://depmap.org/portal/), we obtained methylation level data for the CCNE1 promoter region in a variety of UCEC cell lines [15].

On the online cBioPortal website (https://www.cbioportal.org/), we explored the characteristics of CCNE1 genetic alterations. Based on TCGA tumor datasets in the cBioPortal database, mutation type, alteration frequency, and CNA data were obtained [16]. In the "Comparison/Survival" module, the survival data of all patients providing TCGA tumor samples with or without CCNE1 genetic alterations were also displayed. The difference between groups was considered significant if the log-rank *P* value was less than 0.05. Using the TCGA dataset, we obtained somatic copy number alteration (CNA) and somatic mutation data for UCEC. According to the expression value of CCNE1 in UCEC, patients were divided into two groups based on their CCNE1 expression status: the first 25% of patients were high expression of CCNE1(*n* = 132, CCNE1 ^high^) and the last 25% were low expression of CCNE1(*n* = 132, CCNE1^low^). To download and visualize the somatic mutations in patients with CCNE1^high^ and CCNE1^low^ UCEC across the TCGA datasets, the maftools package was used in R software (https://www.r-project.org/). GISTIC 2.0 (https://cloud.genepattern.org/) was used to evaluate CNAs that were associated with CCNE1 expression and the threshold copy number at alteration peaks [17].

### CCNE1 and its binding protein analysis and m^6^A analysis

Using the STRING (https://www.string-db.org/) and GeneMANIA (http://genemania.org/search/) portals, we analyzed the interactions of the CCNE1 protein with other proteins [18, 19]. In the GeneMANIA portal, there are 20 proteins that can interact with CCNE1 and 103 proteins that can interact with CCNE1 in the String portal. We then further analyzed the interacting proteins of CCNE1 by the Hitpredict portal (http://www.hitpredict.org/) and found that 6 proteins could interact with CCNE1 in all 3 databases. Then, the SRAMP prediction server (http://www.cuilab.cn/sramp) was used to predict m6A modification sites within CCNE1 mRNA [20].

### CCNE1-related gene enrichment analysis

Through the LinkedOmics portal (http://www.linkedomics.org/login.php), CCNE1-related genes were analyzed in UCEC [21]. The top 50 genes positively correlated with CCNE1 and the top 50 genes negatively correlated with CCNE1 in UCEC were obtained via the LinkedOmics portal. In Gene Ontology (GO) and Kyoto Encyclopedia of Genes and Genomes (KEGG) analyses, we explored the biological functions and pathways enriched in CCNE1-related genes. An FDR of 0.05 was chosen as the rank criterion, and 1000 simulations were run.

### Immune cell infiltration analysis

Using the SangerBox portal, the relationships between tumor-infiltrating immune cells (TIICs) and CCNE1 mRNA expression across cancers were explored; the TIIC types included activated CD4 + T cells, activated B cells, activated CD8 + cells, central memory CD8 + T cells, central memory CD4 + T cells, activated dendritic cells, MDSCs, gamma delta T cells, monocytes, macrophages, natural killer cells, type 1 T helper cells and type 17 T helper cells. Then, an investigation of the relationship between CCNE1 expression and ESTIMATE score was conducted for numerous cancers using the SangerBox portal.

Furthermore, using TCGA datasets via SangerBox, we examined the association between CCNE1 mRNA expression level and tumor mutation burden (TMB), microsatellite instability (MSI), neoantigen, and immune checkpoint (ICP) genes expression across cancers. Moreover, the relationships between immune-related cells and CCNE1 expression in UCEC were investigated via ImmuCellAI (http://bioinfo.life.hust.edu.cn/ImmuCellAI#!) online website via TCGA datasets [22]. Using the TIMER (https://cistrome.shinyapps.io/timer/) portal, we further analyzed the correlation between CCNE1 expression and tumor purity, B cell, CD8 + cell, CD4 + cell, macrophage, neutrophil and dendritic cell in UCSC, LUSC, SARC and STAD.

### Cell culture

HEC-1A and HEC-1B are human UCEC cell lines obtained from the American Type Culture Collection. A mycoplasma test was conducted on all cell lines, as well as STR cell identification. MEM (cat.2427827, cat.8122450, Gibco, China) containing 10% fetal bovine serum (FBS) and 1% penicillin was used to culture HEC-1A and HEC-1B cells at 37 °C and 5% CO_2_.

### siRNA delivery, reverse transcription (RT) and quantitative polymerase chain reaction (qPCR)

We obtained CCNE1 small interfering RNAs (siRNAs) from Shanghai GenePharma Co., Ltd. HEC-1A and HEC-1B cells were transfected with siRNAs to knock down CCNE1. Transient transfection of 3 μl si-CCNE1 (20 μM) was performed according to the manufacturer's instructions in 6-well plates via Lipofectamine 3000 (Thermo Fisher Scientific, Inc.). In this study, si-NC was applied as a negative control. Forty-eight hours after transfection, transfected cells were harvested for analysis and detection. The siRNA sequences of CCNE1 were as follows: #1: 5'-UCUGUAUAAAGAUUUGCUGGGTT-3' and #2: 5'-UUCAGAUAUCUGUAAAAGCAATT-3'; the si-NC sequence was sense: 5'- UUCUCCGAACGUGUCACGUTT-3' and antisense: 5'- ACGUGACACGUUCGGAGAATT -3'.

TRIGene (cat. P118-05, GenStar, China) reagent was used to extract total RNA. For cDNA synthesis, 2 μg of RNA was reverse transcribed via a reverse transcription kit (catalog number 00984912, Thermo Fisher Scientific, Inc.). Analysis was performed in triplicate using the SYBR Green reaction mix (cat. B21703, Bimake, USA) on a QuantStudio 3 Real-Time 170 PCR system (Thermo Fisher Scientific). The primer sequences used for RT‒qPCR were as follows: GAPDH-forward: 5’-GGTGGTCTCCTCTGACTTCAACA-3’, GAPDH-reverse: 5’-GTTGCTGTAGCCAAATTCGTTGT-3’; CCNE1-forward: 5’-AGAGGAAGGCAAACGTGACC-3’, CCNE1-reverse: 5’-TATTGTCCCAAGGCTGGCTC-3’.

### Western blotting

The cells in the dish were washed three times in PBS and then lysed using RIPA buffer (cat# R0010, Solarbio, China). A BCA protein assay kit (cat# PC0020, Solarbio, China) was used to measure the protein concentration.

Ten percent sodium dodecyl sulfate PAGE (SDS‒PAGE) was used to load the protein samples, and a PVDF membrane (cat. IPVH00010, Merck Millipove Ltd, Germany) was applied to transfer them. Prior to incubation with primary antibodies overnight at 4 °C, membranes were treated with 5% BSA for two hours at room temperature. The primary antibodies included anti-GAPDH (Proteintech, China) and anti-CCNE1 (Cell Signaling Technology, USA). Then, anti-rabbit or anti-mouse secondary antibodies conjugated with horseradish peroxidase were incubated on the membranes. A Molecular Imager® ChemiDocTM XRS + with Image LabTM Software and an enhanced chemiluminescence kit (cat. WBKLS0100, MILLIPORE, USA) were used to determine the level of protein expression.

### CCK-8 assay

HEC-1A and HEC-1B cells were plated in 96-well plates at 2,000 cells per well, and cells were transfected with CCNE1 siRNAs after 24 h. At specific time points (1, 2, 3, 4 and 5 days), we added 100 μl serum‑free solution (ApexBio) containing 10% CCK-8 reagent (cat. CK04, Dojindo, China) and incubated the samples for 1 h. Then, the OD value was determined using a microplate reader.

### 5-Ethynyl-2'-deoxyuridine (EdU) assay

Two hundred thousand HEC-1A and HEC-1B cells were plated in a 6-well plate after 24 h of siRNA transfection. The EdU kit (cat. K1077, APExBIO, USA) instructions for incubation, fixation, and staining were followed, and imaging analysis was performed using a microscope.

### Transwell assay

Small transwell chambers were seeded with 80,000 UCEC cells with 200 µl of serum-free culture. The wells of the 24-well plate underneath the transwell insert were then filled with 500 µl of serum-containing media. In the invasion experiment, cells were added into each chamber after the mixed matrix gel (Corning Company, USA) (matrix gel:serum-free medium = 1:5) had solidified. The cells were fixed with 4% paraformaldehyde and stained with crystal violet after incubation for 24 h. Furthermore, photographs were obtained under a microscope (BX53, Olympus Company, Japan), and the migrating and invading cells were counted.

### Statistical analysis

The aforementioned online tools were used to automatically perform statistical analyses. To compare two groups, unpaired t tests were employed. Multiple groups were compared using one-way ANOVA, with one exception: the groups in the CCK-8 assay were compared using two-way ANOVA. The results were then subjected to the Bonferroni post hoc test. At *P < 0.05, **P < 0.01, ***P < 0.001 and ****P < 0.0001, these results were declared statistically significant.

## Results

### CCNE1 mRNA expression analysis

**Fig. 1:**
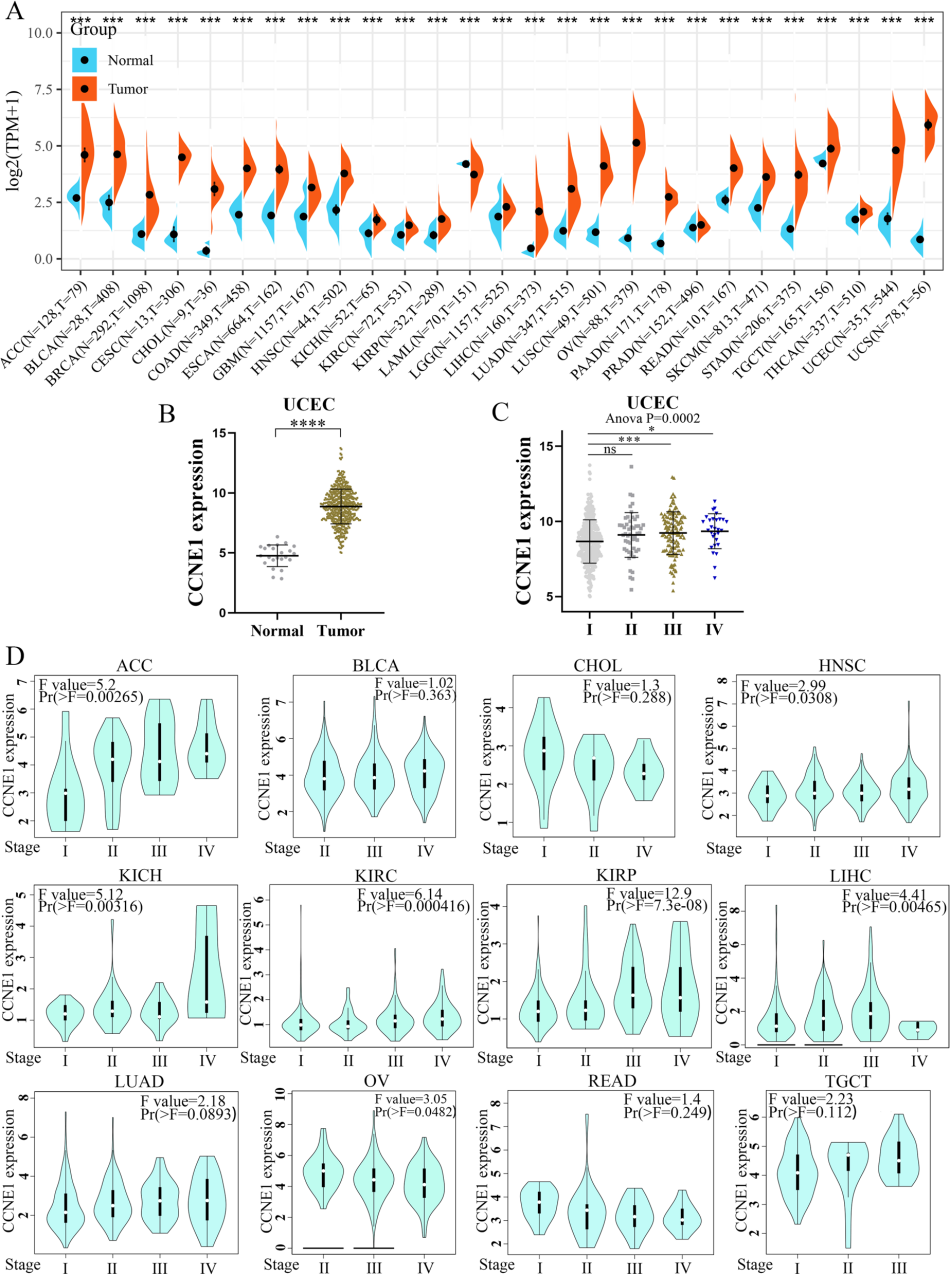
CCNE1 expression levels in normal tissues and tumors including UCEC. **A** CCNE1 expression levels in 27 tumor tissues and normal tissues in TCGA combined with GTEx database via SangerBox. **B** CCNE1 expression levels in UCEC tissues and normal tissues in TCGA. **C** The correlation between CCNE1 expression and UCEC stages (I, II, II and IV) in TCGA datasets. **D** The correlation between CCNE1 expression and tumor stages in various tumors by TCGA cohort. *P < 0.05, ***P < 0.001, ****P < 0.0001

CCNE1 expression in 27 tumors was explored via TCGA and GTEx cohorts, including ACC, BRCA, BLCA, CHOL, CESC, COAD, ESCA, HNSC, GBM, KICH, KIRP, KIRC, LGG, LAML, LUAD, LIHC, LUSC, OV, PRAD, PAAD, READ, STAD, SKCM, THCA, TGCT, UCEC and UCS cohorts, via the SangerBox portal. TCGA and GTEx analyses demonstrated that CCNE1 was highly expressed in multiple tumors, such as ACC, BRCA, BLCA, CHOL, COAD, CESC, GBM, ESCA, HNSC, KIRC, KICH, LGG, KIRP, LUAD, LUSC, LIHC, OV, PRAD, PAAD, STAD, SKCM, READ, THCA, TGCT, UCEC and UCS, while in LAML, the reverse result was significant (Fig. 1A). In UCEC, the CCNE1 expression level was upregulated in tumor tissues compared with normal tissues in the TCGA cohort (Fig. 1B). Additionally, TCGA database analysis showed that high-stage tumors (III-IV) had higher expression levels of CCNE1 than low-stage tumors (I-II) in UCEC (Fig. 1C). The relationships between CCNE1 expression and tumor grade were analyzed via the GEPIA2 portal. CCNE1 expression levels were found to be positively linked with tumor stage in ACC, BLCA, HNSC, KIRC, KICH, KIRP, LIHC, LUAD and TGCT. However, CCNE1 expression was negatively correlated with tumor grade in CHOL, OV and READ (Fig. 1D). These findings revealed that CCNE1 mRNA levels were increased in multiple tumors and that the expression levels of CCNE1 were related to tumor stage in ACC, HNSC, CHOL, KIRP, KIRC, KICH, LIHC, OV, and UCEC.

### Analysis of CCNE1 expression and prognosis

**Fig. 2:**
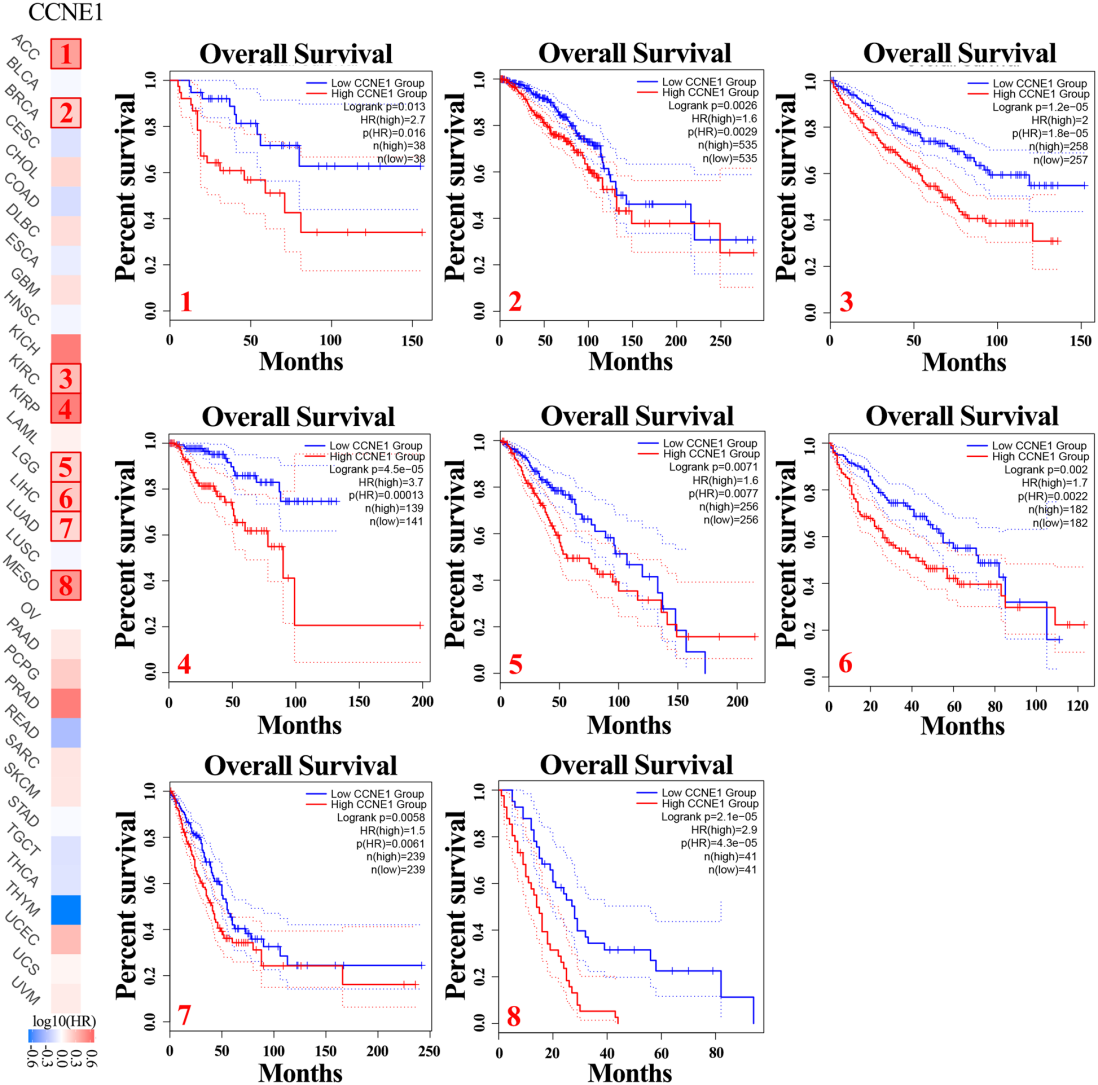
The correlation between CCNE1 expression and overall survival (OS) in various cancers in TCGA database

By comparing the overall survival (OS) and disease-free survival (DFS) rates of tumor patients with low and high CCNE1 expression, the prognostic significance of CCNE1 expression levels in multiple tumors was investigated. By analyzing the pan-cancer cohorts in the GEPIA2 database, we discovered that the expression of CCNE1 was negatively correlated with the OS time of patients in the ACC (HR = 2.7), BRCA (HR = 1.6), KIRC (HR = 2.0), KIRP (HR = 3.7), LGG (HR = 1.6), LIHC (HR = 1.7), LUAD (HR = 1.5) and mesothelioma (MESO) (HR = 2.9) cohorts. This indicated that in these cohorts, patients with high expression levels of CCNE1 showed a poor prognosis (Fig. 2). In addition, the expression of CCNE1 was negatively correlated with the DFS time of patients in BRCA (HR = 1.5), KIRP (HR = 1.8), LGG (HR = 1.8), LIHC (HR = 1.9), MESO (HR = 1.3), PRAD (HR = 2.2), SKCM (HR = 2.0), THCA (HR = 1.8) and UCEC (HR = 1.7) (Figure S1).

Furthermore, CCNE1 expression was revealed as a factor impacting OS in patients with a variety of tumors, including KIRP (HR = 2.59), KIRC (HR = 1.62), MESO (HR = 1.73), KICH (HR = 2.55), LGG (HR = 1.78), ACC (HR = 1.77), LUAD (HR = 1.19), UCEC (HR = 1.23), SKCM (HR = 1.27), PAAD (HR = 1.31) and LIHC (HR = 1.11) (Figure S2A), by Cox analysis. Using the Kaplan‒Meier Plotter portal, the correlation between CCNE1 mRNA expression and the prognosis of patients with ovarian cancer, breast cancer, gastric cancer lung cancer, and liver cancer was investigated. According to the results, patients with high CCNE1 expression had a poorer prognosis than those with low expression in the ovarian cancer, breast cancer, gastric cancer, lung cancer, and liver cancer cohorts (Figure S2B). Through TCGA database analysis, we discovered that CCNE1 expression level was negatively related to prognosis in patients with UCEC (Figure S2C). These findings revealed that high CCNE1 mRNA levels were linked to a poor prognosis in various cancers, including UCEC.

### Analysis of CCNE1 DNA methylation

**Fig. 3:**
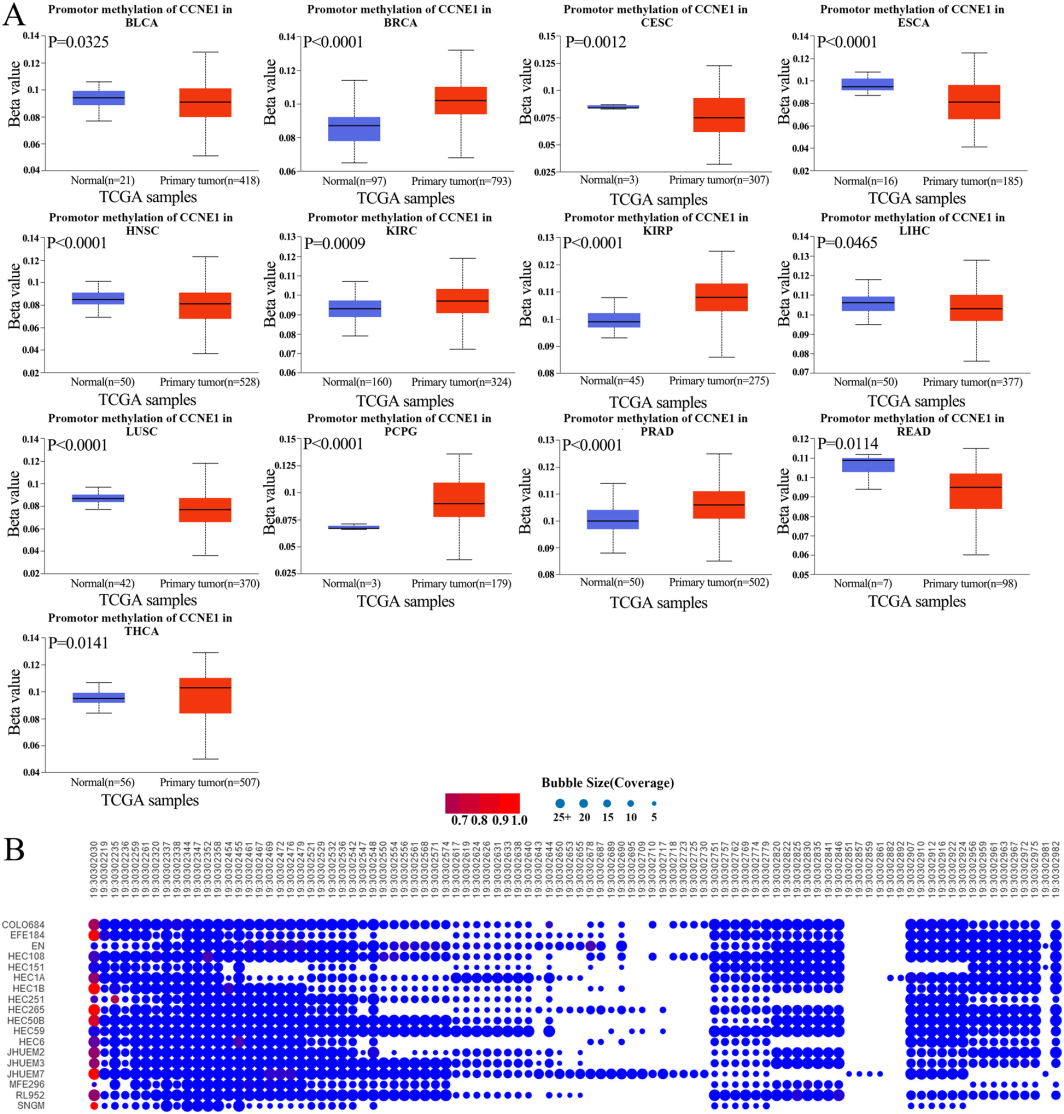
DNA promotor methylation levels of CCNE1 in pan-cancers. **A** The DNA promotor methylation levels of CCNE1 in various cancers and normal tissues in TCGA database. **B** The DNA promotor methylation levels of CCNE1 in UCEC cell lines

Oncogenesis and abnormal methylation are related, and the methylation patterns of tumors and normal tissues are different [23]. Methylation can either encourage or prevent the growth of tumors [24]. Using the UALCAN online portal, methylation levels at the CCNE1 promoter region were investigated across cancers. Compared to those in corresponding normal samples, the promoter methylation levels of CCNE1 were lower in BLCA, ESCA, CESC, HNSC, LIHC, LUSC and READ samples and higher in BRCA, KIRP, KIRC, PCPG, PRAD and THCA samples (Fig. 3A). Based on these findings, it appears that methylation of the CCNE1 promoter may influence its expression in a variety of cancers. We found that the methylation level of the CCNE1 promoter region was low in a variety of UCEC cell lines, suggesting that a low methylation level of the CCNE1 promoter may be associated with abnormally high CCNE1 expression in UCEC (Fig. 3B).

### Analysis of CCNE1 genetic alterations in different cancers, including UCEC

**Fig. 4:**
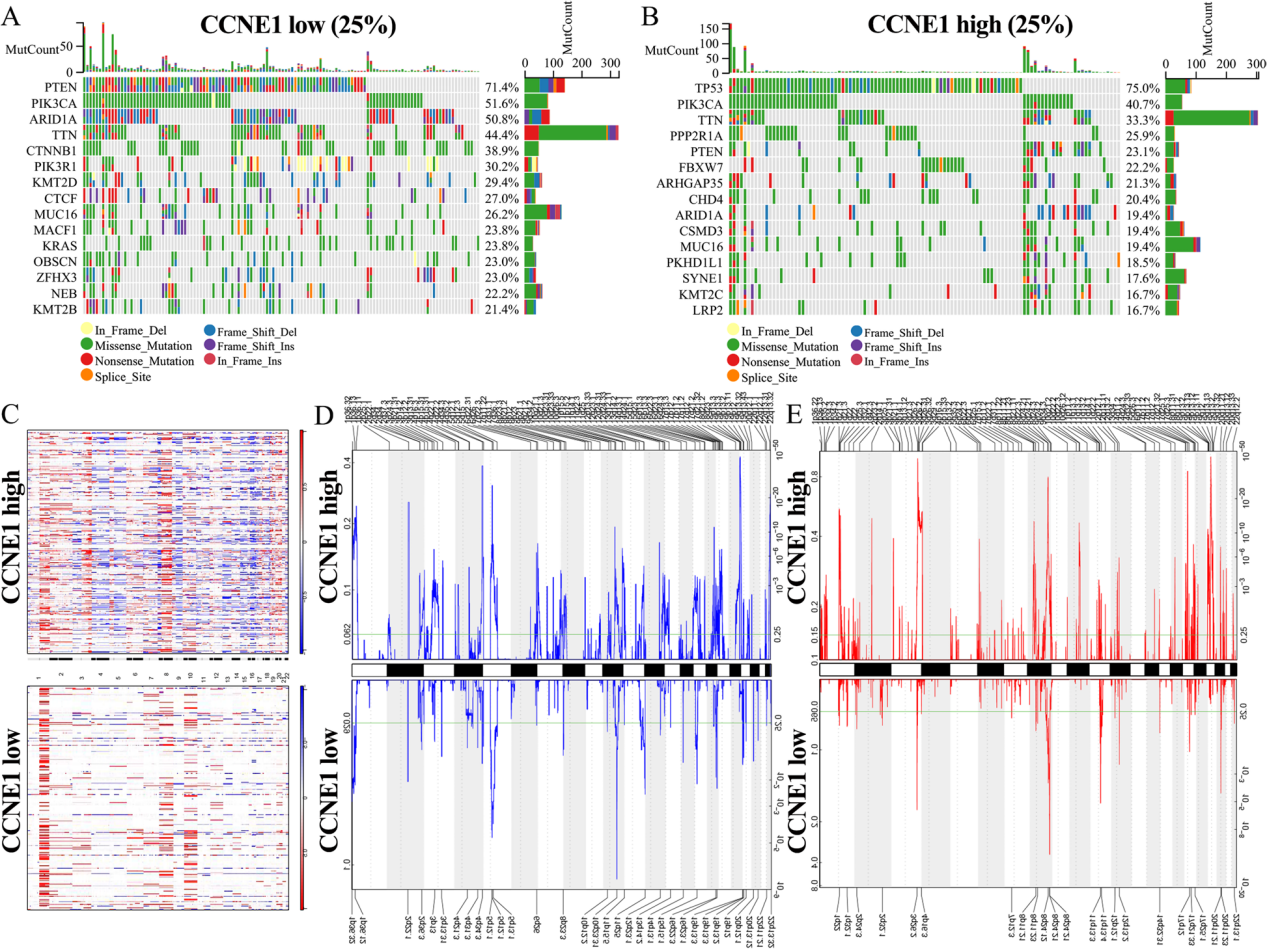
Distinct genomic profiles associated with CCNE1 expression. Detection of differential somatic mutations in UCEC, including 25% CCNE1^low^ group (**A**) and 25% ALKBH5^high^ group (**B**). **C** The CNAs profile analysis about 25% CCNE1^low^ group and 25% CCNE1.^high^ group in TCGA dataset by GISTIC2.0. (D-E) Frequency of amplifications and deletions in UCEC with CCNE1 low or high expression (red, amplification, Blue, deletion)

Tumorigenesis and progression are correlated with mutations, amplifications, or deletions of oncogenes or tumor suppressor genes [25]. Using cBioPortal, we comprehensively examined diverse types of modifications in the CCNE1 gene, such as mutations, amplifications, deep deletions and structural variants. It was found that amplification was the most frequent type of genetic alteration in the CCNE1 gene in UCS (40.35%), OV (19.01%) and STAD (10.91%); mutation (4.16%) was the most common type in UCEC, and deep deletion (1.15%) was the most common type in MESO (Figure S3A). Then, we explored the genetic alterations of CCNE1 in various tumors. Based on the results, missense mutations appeared to be the predominant genetic alteration of CCNE1 (Figure S3B). Figure S3C shows the 3D structure of CCNE1. In addition, the relationship between CCNE1 gene alteration status and prognosis in various cancers was explored via cBioPortal analysis of the TCGA dataset. The findings suggested that patients with tumors without CCNE1 alterations had better OS and DFS than patients with tumors with CCNE1 alterations (Figure S3D-E). Moreover, the correlations between CCNE1 expression and somatic mutations or copy number variations (CNVs) were explored via the TCGA-UCEC cohort. The CCNE1^high^ group (*n* = 132) displayed a high frequency of somatic mutations in the TP53 (75%), PIK3CA (40.7%), TTN (33.3%), PPP2R1A (25.9%) and PTEN (23.1%) genes, and the CCNE1^low^ group (*n* = 132) displayed a high frequency of mutations in the PTEN (71.4%), PIK3CA (51.6%), ARID1A (50.8%), TTN (44.4%) and CTNNB1 (38.9%) genes (Fig. 4A-B). Figure 4C-E shows the comparison of the CNV profiles in the CCNE1^low^ (*n* = 132) and CCNE1^high^ (*n* = 132) groups. In the CCNE1^low^ group, we discovered amplification peaks of chromosomal locations in 3q26.2, 8q24.12, 8q24.21, 11q13.3 and 12q13.2 and frequent deletions of chromosomal regions in 1p36.32, 2q22.1, 5q12.1, 10q23.31, 11q25, 15q15.1 and 16q22.3 (Fig. 4C-E). In the CCNE1^high^ group, we discovered amplification peaks of chromosomal regions in 1q21.3, 3q26.2, 8q24.21, 10q22.3, 17q11.2 and 19q12 and deletions of chromosomal regions in 1p36.11, 2q22.1, 4q35.2, 5q12.3, 10q23.31, 16q22.3, 19p13.3 and 22q13.32 (Fig. 4C-E). According to these results, various tumors exhibited mutations, amplifications, and deletions of the CCNE1 gene. Most CCNE1 gene mutations in the pancancer dataset were missense mutations. Furthermore, the UCEC samples of the TCGA dataset showed diverse CNVs and somatic mutations when grouped by CCNE1 expression level. The results showed that gene alterations of CCNE1 may regulate the growth and progression of a variety of tumors, including UCEC.

### The interactions between CCNE1 and its binding proteins

**Fig. 5:**
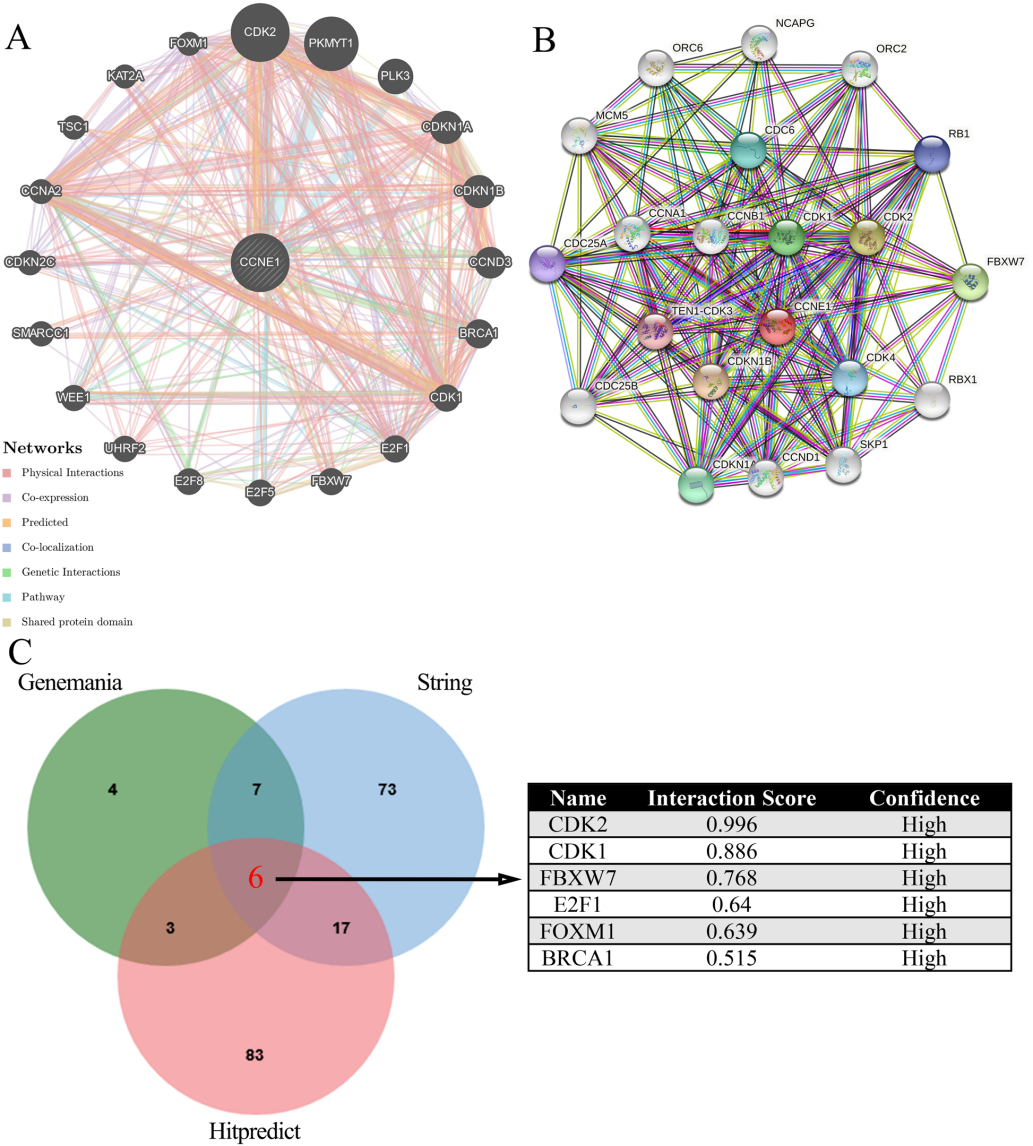
The interactions between CCNE1 and its binding proteins. The interacting proteins of CCNE1 in the Genemania (**A**) and STRING (**B**) portals. **C** The intersection of CCNE1 binding proteins in Genemania, STRING and Hitpredict portals, including CDK2, CDK1, FBXW7, E2F1, FOXM1 and BRCA1

CCNE1 has been reported to interact with a variety of proteins to function as an oncogene in a variety of tumors [6, 9]. Through the STRING and Genemania analyses, we found the interacting protein of CCNE1. Figure 5A-B showed the interacting protein networks of CCNE1 protein in STRING and Genemania. 20 and 103 proteins were enriched in these two interacting protein networks, respectively, and 13 proteins were enriched in both networks, including BRCA1, CCNA2, CCND3, CDK1, CDK2, CDKN1A, CDKN1B, CDKN2C, E2F1, FBXW7, FOXM1, PKMYT1 and WEE1 (Fig. 5C). Then, we used Hitpredict database to further analyze the interacting proteins of CCNE1 and obtained 6 proteins that strongly interacted with CCNE1 by intersections with the proteins in both the STRING and Genemania portal, including CDK2, CDK1, FBXW7, E2F1, FOXM1 and BRCA1 (Fig. 5C). The results showed that CCNE1 could combine with CDK2, CDK1, FBXW7, E2F1, FOXM1 and BRCA1 to regulate the biological functions of tumors.

### The interactions between CCNE1 and the m^6^A modification of CCNE1 mRNA

**Fig. 6:**
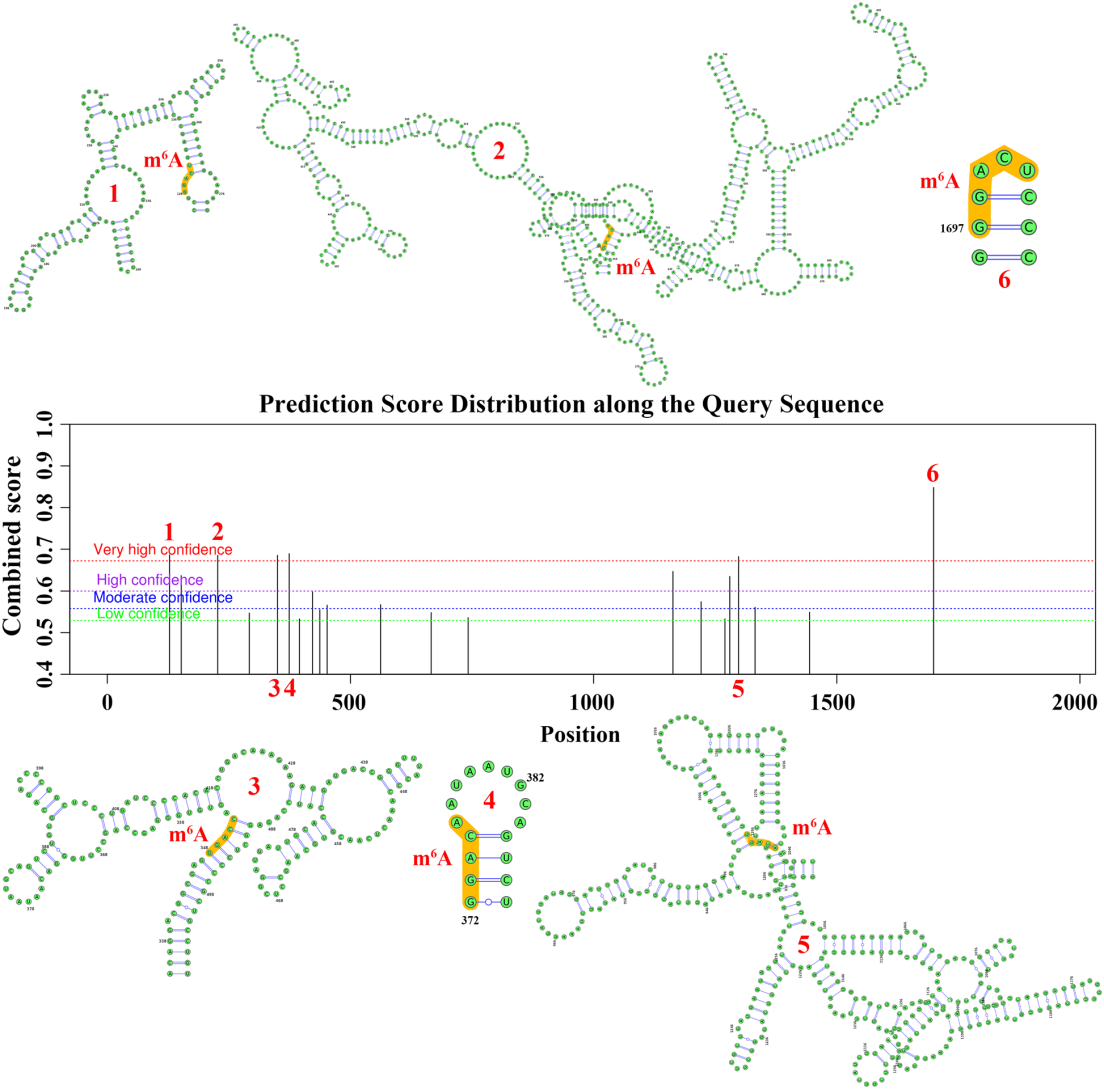
The m6A modification of CCNE1 mRNA via SRAMP portal

As an important regulatory factor in various physiological processes and disease progression, posttranscriptional modification has received increasing attention in the biological sciences. Among the various RNA modifications, N6-methyladenosine (m6A) is the most abundant. Numerous studies have reported that m6A modification plays a crucial role in numerous types of cancer, so we explored whether CCNE1 is affected via m6A modification. Figure 6 shows that CCNE1 may have six functional areas that can be modified by m6A with very high confidence according to the SRAMP portal. These results showed that the biological function of CCNE1 in various tumors may be affected by m6A modification (Fig. 6).

### Analysis of CCNE1-related genes in UCEC

**Fig. 7:**
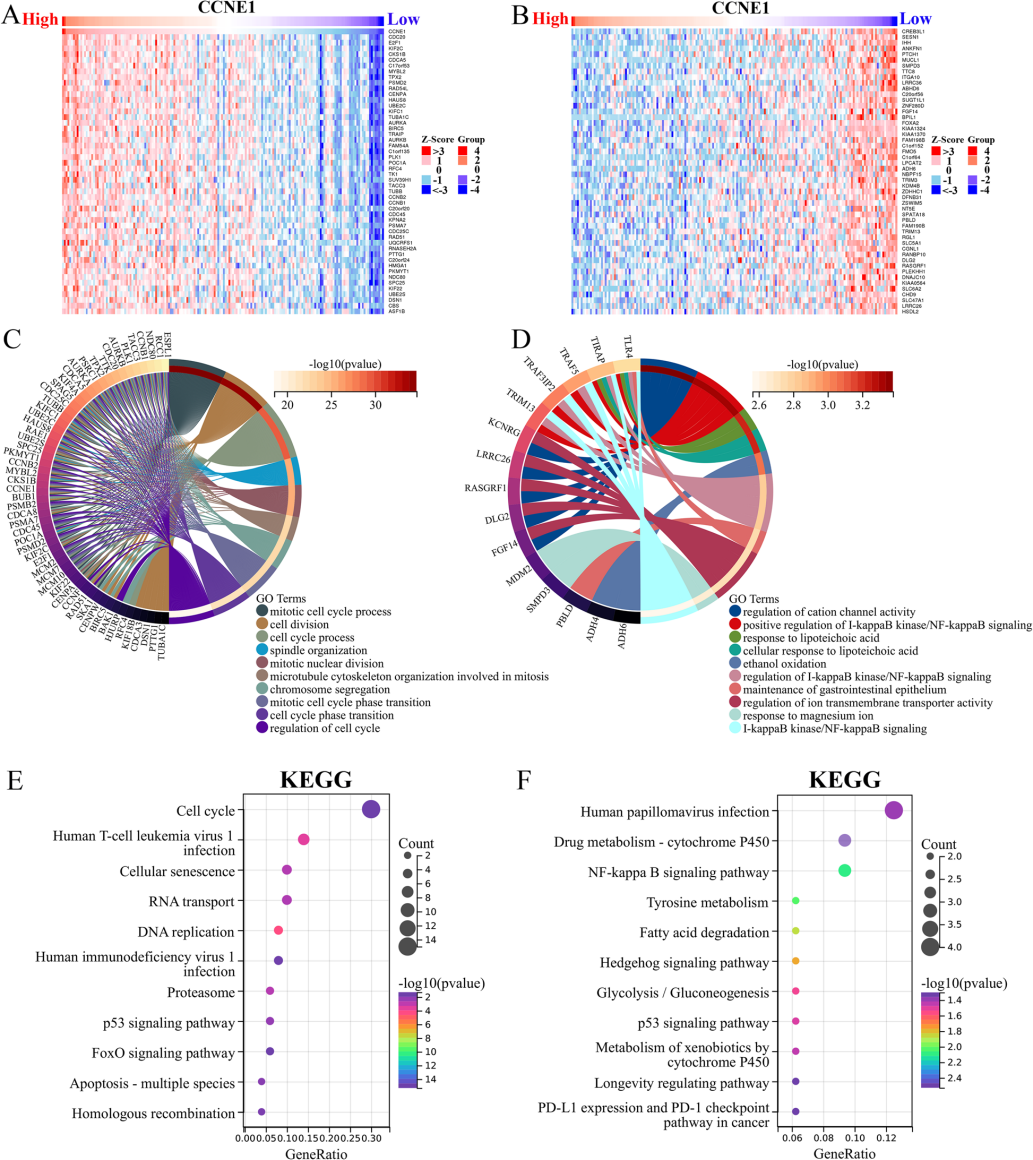
The analyses of CCNE1-related genes in UCEC. Heatmaps showing the enrichment patterns of the top 100 genes positively (50) (**A**) or negatively (50) (**B**) correlated with CCNE1 expression of UCEC in TCGA database. The GO-BP enrichment analysis were applied with the top 50 CCNE1 positive (**C**) or negative (**D**) related genes. The KEGG enrichment analysis were used to analyze the top 50 CCNE1 positive (**E**) or negative (**F**) related genes

Using the LinkedOmics portal, we analyzed CCNE1-related genes to investigate the role of the CCNE1 gene in UCEC tumor development. We identified the top 50 genes positively correlated with CCNE1 (Fig. 7A) and the top 50 genes negatively correlated with CCNE1 (Fig. 7B) in UCEC. The GO analysis showed that the top 50 genes positively correlated with CCNE1 were enriched in cell division, mitotic cell cycle process, cell cycle process, spindle organization and mitotic nuclear division, and the top 50 genes negatively correlated with CCNE1 were enriched in regulation of cation channel activity, response to lipoteichoic acid and positive regulation of I-kappaB kinase/NF-kappaB signaling (Fig. 7C-D). Figure 7E-F displays the Kyoto Encyclopedia of Genes and Genomes (KEGG) enrichment analysis results for CCNE1-related genes in UCEC. The top 50 genes positively related to CCNE1 were enriched in the cell cycle, RNA transport, P53 signaling pathway, DNA replication and FoxO signaling pathway (Fig. 7E). The top 50 genes negatively related to CCNE1 were enriched in drug metabolism-cytochrome P450, NF-kappaB signaling pathway, fatty acid degradation, hedgehog signaling pathway and p53 signaling pathway (Fig. 7F). These findings demonstrated that CCNE1 could regulate cell cycle-, metabolism- and DNA repair-associated signaling pathways in UCEC.

### CCNE1 expression and immunity in across cancers, including UCEC

**Fig. 8:**
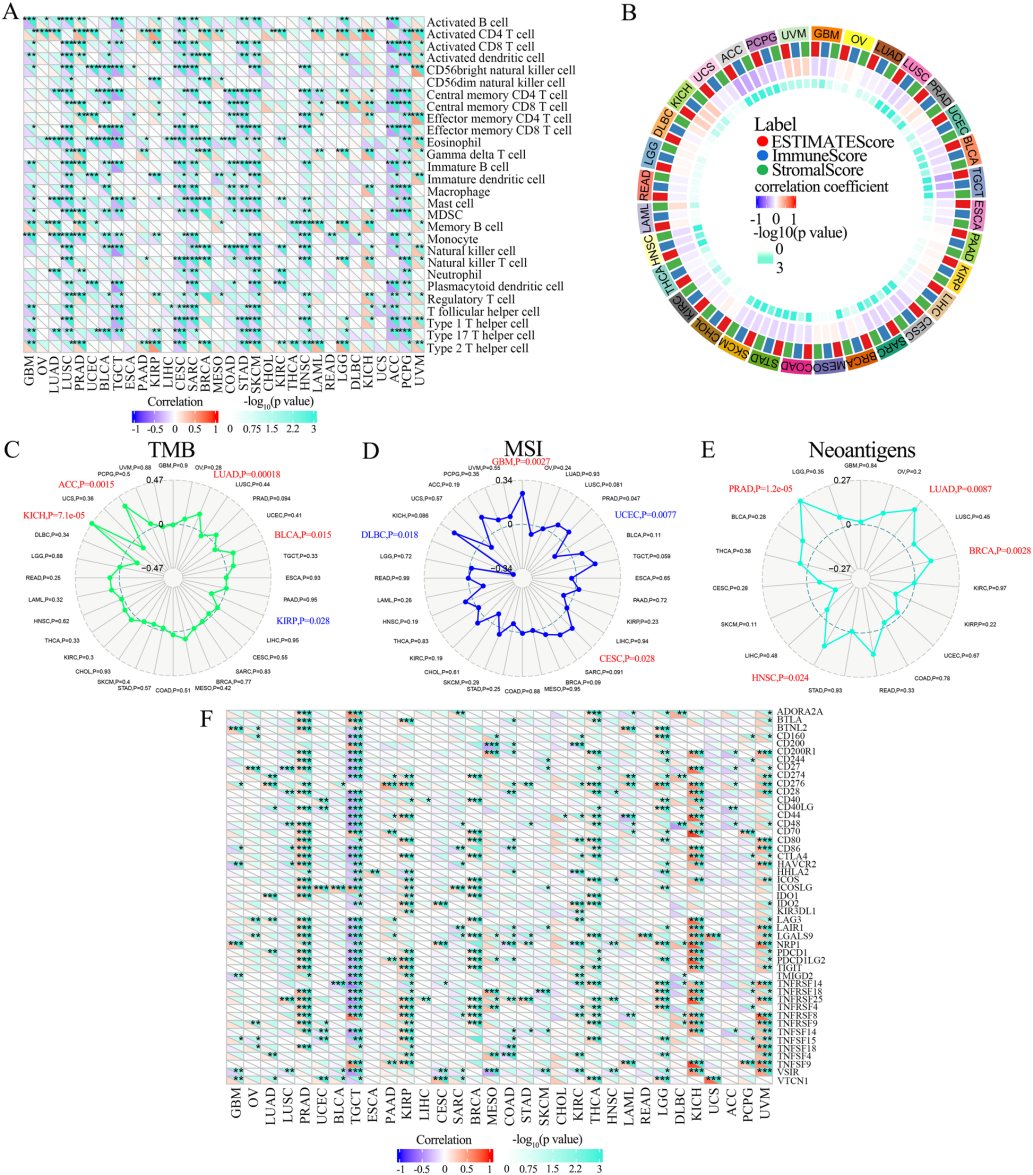
Correlations between CCNE1 mRNA expression and immune infiltration. **A** The correlations between CCNE1 expression and immune cell infiltration levels in various tumors through Sangerbox. **B** The correlations between ESTIMATE scores (ESTIMATE Score, Immune Score, and Stromal Score) and CCNE1 expression in various tumors via Sangerbox portal. The relationships between ccne1 mRNA expression and TMB (**C**), MSI (**D**), neoantigen (**E**) and ICP-gene (**F**) in multiple cancers. *P < 0.05, **P < 0.01, ***P < 0.001, ****P < 0.0001

Numerous studies have claimed that tumor-infiltrating immune cells (TIICs) play a crucial role in tumor development and progression. As important elements of the tumor immune microenvironment (TIM), TIICs play a significant role in the development, metastasis, and growth of tumors [26, 27]. To examine the correlation between TIICs and CCNE1 expression across cancers, we analyzed the correlation between CCNE1 and the composition of TIICs in various tumors. First, we used the Sangerbox portal to investigate the connection between TIIC levels and CCNE1 expression in multiple tumors. These results demonstrated that CCNE1 expression was negatively correlated with the infiltration of various types of immune cells in GBM, LUSC, BLCA, TGCT, CESC, SARC, COAD, STAD, SKCM, HNSC, ACC, and PCPG and positively correlated with the infiltration of immune cells in PRAD, LGG, LAML, KICH and UVM (Fig. 8A). Moreover, we evaluated the association between CCNE1 and ESTIMATE scores (immune, stromal and ESTIMATE scores) in tumors. The percentage of stromal cells in tumor tissues is indicated by the stromal score, the percentage of immune cells infiltrating the tumor tissues is indicated by the immune score, and tumor purity is indicated by the ESTIMATE score, which is the sum of the stromal and immune scores. The results suggested a negative correlation between CCNE1 and the immune, stromal and ESTIMATE scores in ACC, TGCT, SARC, STAD and SKCM and a positive correlation between CCNE1 and the immune, stromal and ESTIMATE scores in KICH and UVM (Fig. 8B). This demonstrated that increased CCNE1 expression was associated with limited stromal and immune cell infiltration, leading to high tumor purity in ACC, TGCT, SARC, STAD, and SKCM.

It has been demonstrated that tumor mutation burden (TMB), microsatellite instability (MSI), and neoantigen are related to antitumor immunity, which is a powerful indicator of the effectiveness of immunotherapy for tumors [28]. TMB and MSI-high (MSI-H) cancers respond well to immune checkpoint inhibitors, and altered tumor-specific antigens, known as neoantigens, are intriguing targets for tumor immunotherapy using T cells [29, 30]. We explored the association between the expression levels of CCNE1 and TMB, MSI, or neoantigen to investigate whether CCNE1 acts as an indicator of immunotherapeutic responses across cancers. Figure 8C demonstrates that CCNE1 expression was positively associated with TMB in ACC, BLCA, LUAD and KICH. CCNE1 expression had a positive correlation with MSI in GBM, PRAD and CESC and a negative correlation in UCEC and DLBC (Fig. 8D). CCNE1 expression was positively correlated with neoantigen in LUAD, BRCA, HNSC and PRAD (Fig. 8E).

Since immune checkpoint (ICP) blockade proteins control immune cell infiltration into the tumor microenvironment, they are potentially targeted for cancer immunotherapeutic therapies [31]. Subsequently, we explored the relationships between CCNE1 and ICP expression in various cancers. CCNE1 expression had a positive association with ICP genes in PRAD, KIRP, BRCA, KIRC, THCA, LGG, KICH, and UVM and a negative correlation with ICP genes in TGCT (Fig. 8F). Especially in KICH and UVM, there was a strongly positive relationship between CCNE1 and ICP gene expression. These findings revealed that CCNE1 influenced PRAD, KIRP, BRCA, KIRC, THCA, LGG, KICH, and UVM sensitivity to immune checkpoint inhibitor therapies, and patients with high levels of CCNE1 expression may have a poor response to immunotherapies targeting genes involved in ICP in TGCTs. Moreover, we further investigated the link between CCNE1 expression and TIICs in UCEC with the TCGA dataset via ImmuCellAI. These findings suggested that the expression level of CCNE1 was positively related to the infiltration of DCs, macrophages and gamma delta cells and negatively related to the infiltration of NK, CD4 T, CD4 naïve, Tr1, iTreg, nTreg, Th2, Th17, Tfh and central memory cells (Figure S4A-B). Moreover, we further analyzed the correlation between CCNE1 expression and tumor purity, B cell, CD8 + cell, CD4 + cell, macrophage, neutrophil and dendritic cell (DC) in UCSC, LUSC, SARC and STAD via the TIMER portal. The results showed that CCNE1 expression level was negatively correlated with the infiltration of CD8 + T cell, macrophage and DC cell, and positively correlated with the infiltration of neutrophil in UCEC (Figure S5A). In LUSC, CCNE1 expression was negatively correlated with the infiltration of B cell, CD8 + T cell, macrophage, neutrophil and DC cell (Figure S5B). In SARC, CCNE1 expression was negatively related to the infiltration of CD4 + T cell (Figure S5C). In STAD, CCNE1 expression was negatively associated with the infiltration of B cell, CD8 + T cell, CD4 + T cell, macrophage, neutrophil and DC cell (Figure S5D). As a result, CCNE1 mediates the regulation of ICP genes and acts as a promising target for immunotherapy for tumors including UCEC.

### CCNE1 promotes the proliferation, migration and invasion of UCEC cells

**Fig. 9:**
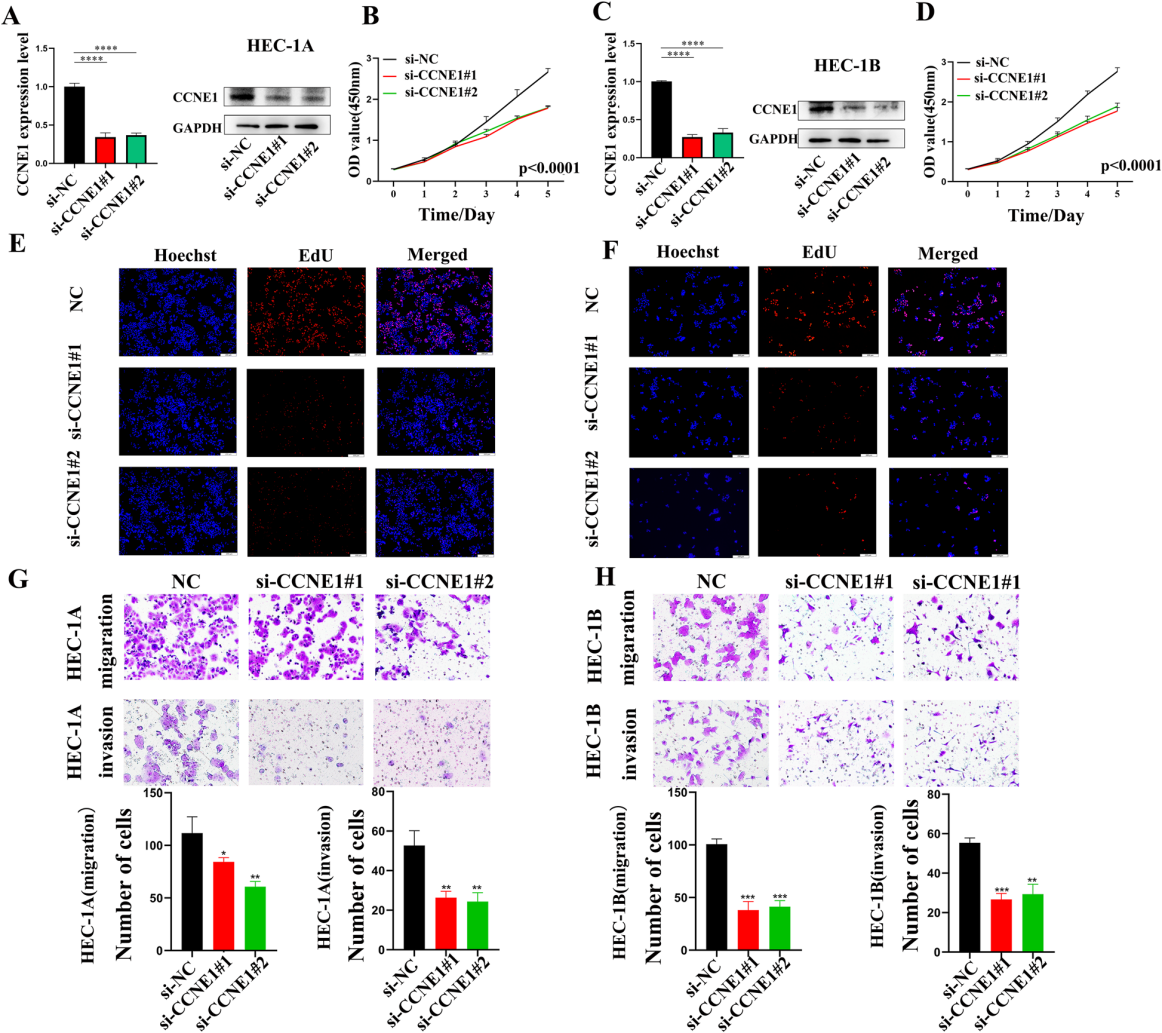
The biological functions of CCNE1 inUCEC. Verification of knockdown efficiency of CCNE1 in HEC-1A (**A**) and HEC-1B (**C**) cell lines via RT-qPCR and western blot. The biological functions of CCNE1 on UCEC cell lines were verified by CCK-8 (**B** and **D**), EdU (**E–F**) and Transwell (**G-H**) experiments. *P < 0.05, **P < 0.01, ***P < 0.001, ****P < 0.0001

To confirm the bioinformatics results, experimental verification of the results of the functional enrichment analysis was needed. Proliferation, migration, and invasion experiments were conducted on two classical UCEC cell lines, HEC-1A and HEC-1B. The expression of CCNE1 in HEC-1A and HEC-1B cell lines was knocked down using siRNA. Western blotting and RT‒qPCR were used to confirm the efficiency of CCNE1 knockdown (Fig. 9A and C). CCNE1 knockdown significantly reduced cell proliferation as determined by CCK-8 and EdU assays (Fig. 9B, D and E–F). Furthermore, CCNE1 knockdown significantly reduced the migration and invasion abilities of HEC-1A and HEC-1B cells (Fig. 9G-H). Thus, the role of CCNE1 in the regulation of cell proliferation, migration and invasion in the development of UCEC was experimentally validated.

## Discussion

The overexpression of CCNE1 has been well documented in a variety of human tumors, including breast, ovarian, liver and lung cancers [6–10]. It is thought that CCNE1 overexpression results in ineffective DNA replication, premature entry into S-phase, and genomic instability and is essential for tumor cell proliferation. Human cancers frequently express cell cycle regulators at high levels, which conceivably could be a result of a higher mitotic index [7, 8].

In our research, we explored whether the mRNA expression of CCNE1 was increased in multiple tumors compared with normal tissues, including ACC, BRCA, BLCA, CHOL, COAD, CESC, ESCA, HNSC, GBM, KIRC, KIRP, KICH, LGG, LUAD, LUSC, LIHC, OV, PRAD, PAAD, READ, STAD, SKCM, THCA, TGCT, UCEC and UCS. This suggested that CCNE1 may play an oncogene role in these tumors, driving the occurrence and development of these tumors. The levels of CCNE1 expression were shown to be significantly associated with tumor grade in UCEC and tumor stage across cancers, including ACC, HNSC, KIRC, KIRP, KICH and LIHC. Then, prognosis analysis demonstrated that high mRNA levels of CCNE1 were associated with poor OS in ACC, BRCA, KIRC, KIRP, LGG, LIHC, LUAD and MESO. Cox analysis also showed that CCNE1 expression was a factor influencing OS in patients with multiple types of cancers, including KIRP, KIRC, MESO, KICH, LGG, ACC, LUAD, UCEC, SKCM, PAAD and LIHC. These findings revealed that high CCNE1 expression was related to a poor prognosis in numerous cancers, including UCEC. Zhu et al. found that SENP1 could promote STC1 expression and upregulated CCNE1 through driving the small ubiquitin-like modifier (SUMO)ylation of HIF-1α, which facilitated the malignant phenotypes of Wilms tumor cells [32]. And Ma et al. revealed that CCNE1 can promote progression and is associated with poor prognosis in lung adenocarcinoma [33]. These findings further suggested that CCNE1 has great potential to be developed as a predictor of tumor prognosis.

Moreover, the methylation levels of the CCNE1 promoter were lower in BLCA, CESC, HNSC, ESCA, LUSC, LIHC and READ and higher in BRCA, KIRP, KIRC, PRAD, PCPG and THCA than in corresponding normal tissues, suggesting that CCNE1 promoter methylation may lead to its downregulation or upregulation in various tumor tissues and that low methylation levels of the CCNE1 promoter may be related to abnormally high CCNE1 expression in UCEC. GO and KEGG enrichment analyses of CCNE1-related genes demonstrated that CCNE1 expression was significantly associated with to the cell cycle, metabolism and DNA damage repair in UCEC patients. Additionally, patients with tumors free of CCNE1 mutations had better OS prognoses than patients with modifications, indicating that CCNE1 may have an oncogenic function in various tumors. This further indicated that CCNE1 acted as an oncogene in a variety of tumors and has the potential to be a indicator of tumor prognosis.

Consequently, TIICs play an important role in the development, progression, and management of cancers as a prominent part of the immune microenvironment [26, 27]. There were significant correlations between CCNE1 mRNA and several TIICs across cancers, including UCEC. In PRAD, LAML, LGG, KICH and UVM, there was a positive association between CCNE1 mRNA expression and TIICs, whereas in GBM, LUSC, BLCA, TGCT, CESC, SARC, COAD, STAD, SKCM, HNSC, ACC, and PCPG, there was a negative correlation. The impacts of CCNE1 on the immunotherapy sensitivity of cancer patients were then evaluated. According to TMB, MSI, neoantigen, and ICP analyses, CCNE1 may be a potential therapeutic target, particularly for immunotherapy, for a variety of tumor types. In vitro assays also revealed that CCNE1 knockdown reduced the proliferation and invasion abilities of HEC-1A and HEC-1B cells. On the basis of these findings, CCNE1 was hypothesized to be a factor that promotes the development and progression of multiple cancers, particularly UCEC.

## Conclusion

These findings suggest that CCNE1 may be a crucial prognostic marker and a promising indicator of immunotherapy sensitivity in individuals with malignant tumors, including UCEC. In particular, CCNE1 knockdown suppressed the malignant phenotype of UCEC. CCNE1 may be a potential therapeutic target in UCEC.

## Supplementary Information

**Additional file 1.** Supplementary figures.

## Data Availability

The datasets used and/or analyzed during the present study are available from the corresponding author on reasonable request.

## Abbreviations

ACC: Adrenocortical carcinoma
BLCA: Bladder urothelial carcinoma
BRCA: Breast invasive carcinoma
CESC: Cervical squamous cell carcinoma and endocervical adenocarcinoma
CHOL: Cholangiocarcinoma
COAD: Colon adenocarcinoma
ESCA: Esophageal carcinoma
GBM: Glioblastoma multiforme
HNSC: Head and neck cancer
KICH: Kidney chromophobe
KIRC: Kidney renal clear cell carcinoma
KIRP: Kidney renal papillary cell carcinoma
LAML: Acute myeloid leukemia
LGG: Brain lower grade glioma
LIHC: Liver hepatocellular carcinoma
LUAD: Lung adenocarcinoma
LUSC: Lung squamous cell carcinoma
OS: Overall survival
OV: Ovarian serous cystadenocarcinoma
PAAD: Pancreatic adenocarcinoma
PRAD: Prostate adenocarcinoma
READ: Rectum adenocarcinoma
SKCM: Skin cutaneous melanoma
STAD: Stomach adenocarcinoma
TGCT: Testicular germ cell tumors
THCA: Thyroid carcinoma
UCEC: Uterine corpus endometrial carcinoma
UCS: Uterine carcinosarcoma
