# Supplementary material for: CCNE1 is a predictive and immunotherapeutic indicator in various cancers including UCEC: a pan-cancer analysis
Source: Hereditas. 2023 Mar 24;160:13. doi: 10.1186/s41065-023-00273-0 (PMC10037856; doi:10.1186/s41065-023-00273-0)
Supplement: Supplementary file 1 — Additional file 1. Supplementary figures. [file 41065_2023_273_MOESM1_ESM.docx]

Supplementary materials


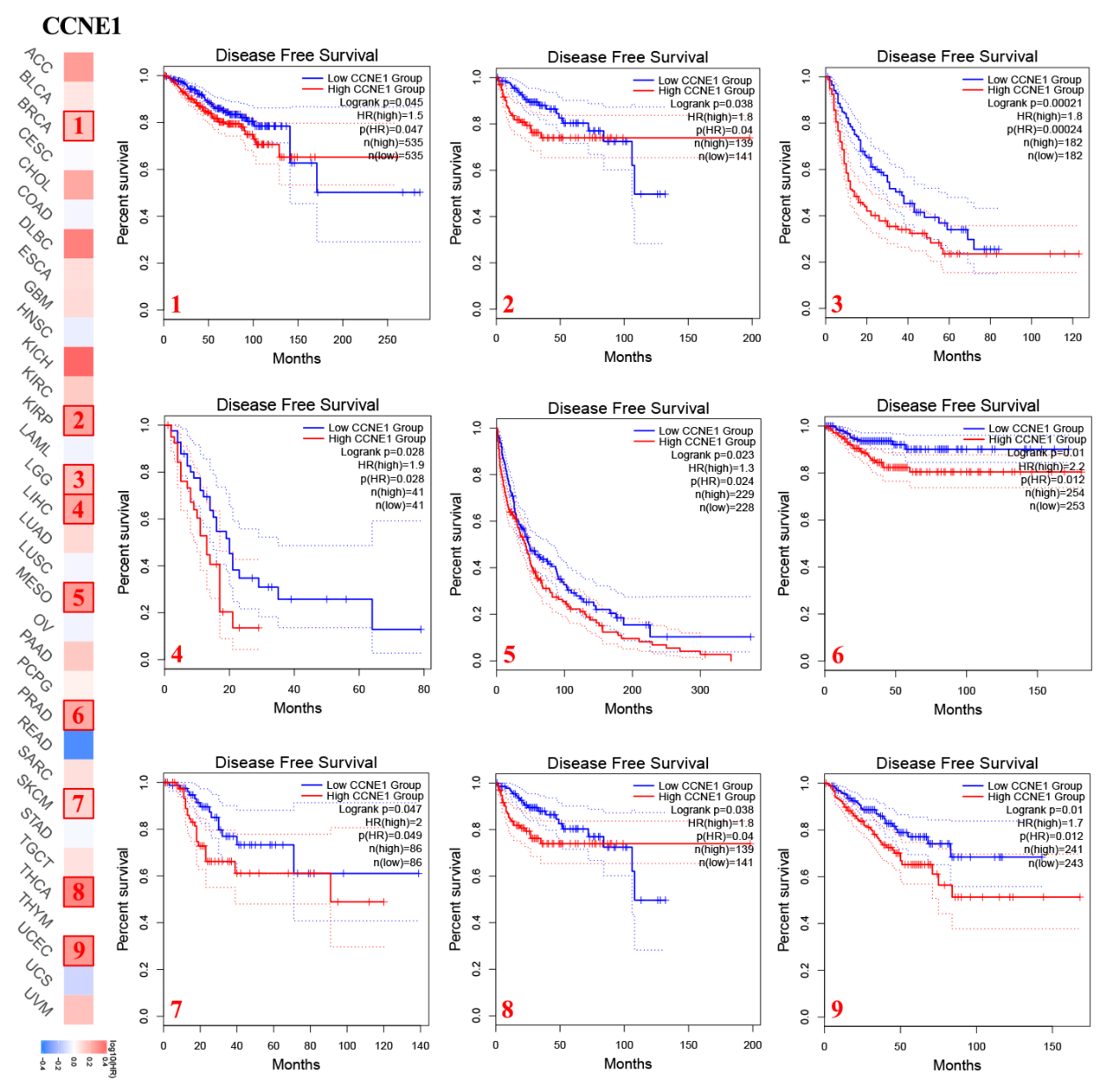


**Supplementary Figure 1. The correlation between CCNE1 expression and disease-free survival (DFS) in various cancers in TCGA database.**


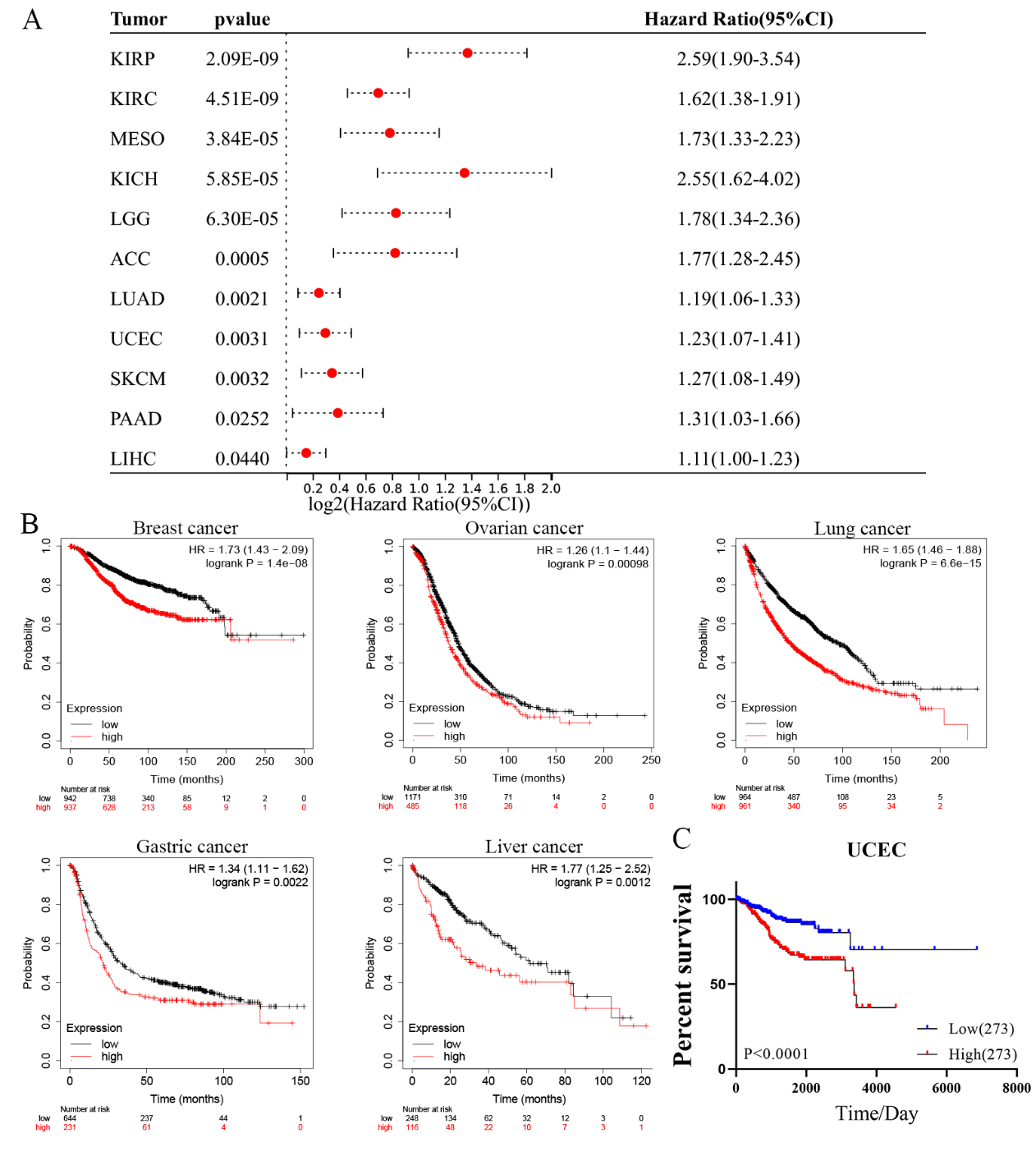


**Supplementary Figure 2. The correlation between CCNE1 expression and prognosis in various tumors via TCGA. (A)** The relationships between CCNE1 expression and OS prognosis of various cancers via SangerBox. **(B)** Correlations between the expression level of the CCNE1 and OS in breast cancer, ovarian cancer, lung cancer, gastric cancer and liver cancer by Kaplan-Meier plotter portal. **(C)** The correlation between CCNE1 expression and OS in UCEC by TCGA database.


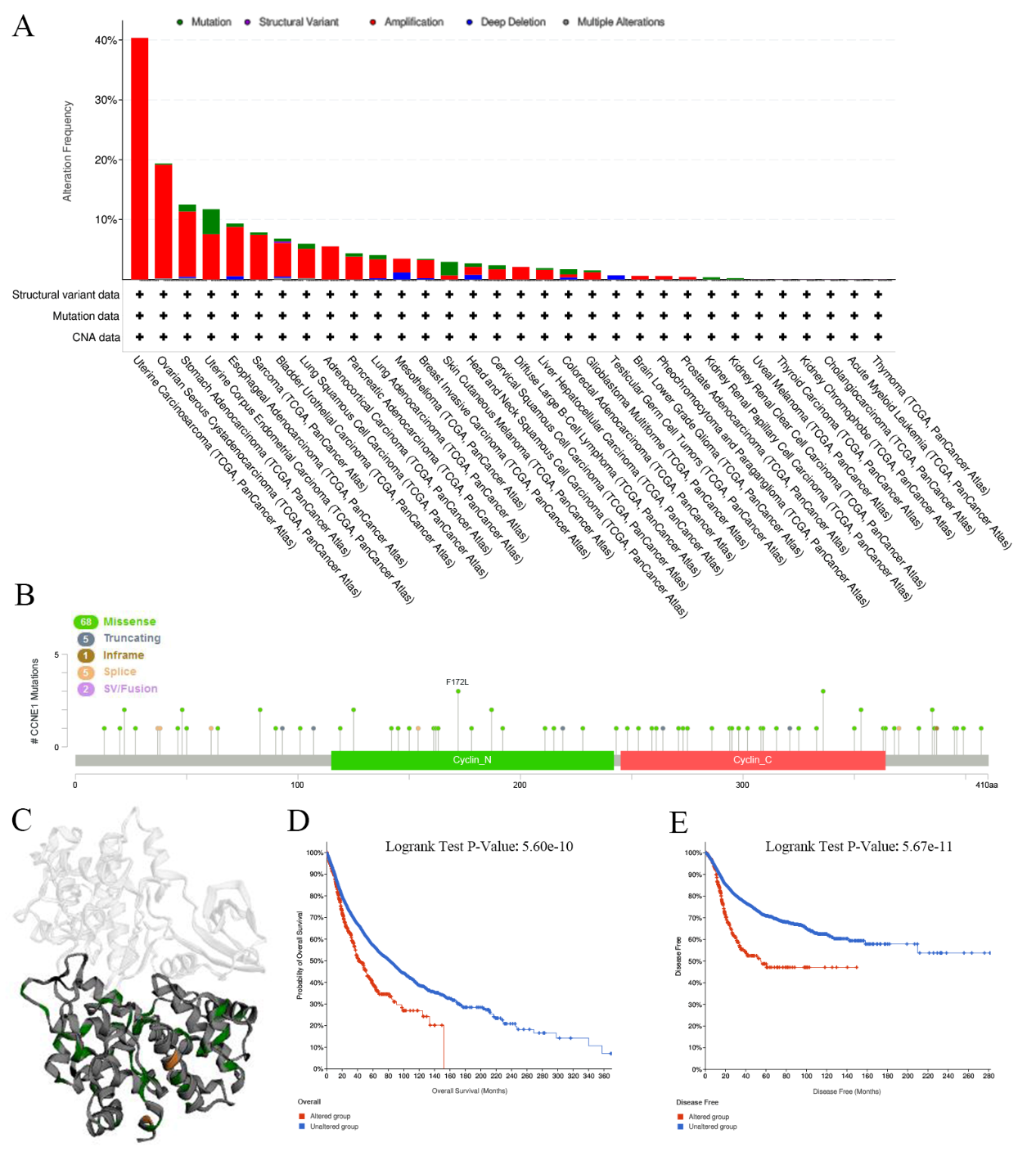


**Supplementary Figure 3. Mutation feature of CCNE1 in pan-cancers via the cBioPortal tool. (A)** The alteration frequency with mutation type **(A)** and mutation site **(B)** displayed in various tumors. **(C)** The protein structure of CCNE1. The potential correlation between CCNE1 mutation status and overall survival **(D)** or disease-free survival **(E)** of all TCGA tumors using the cBioPortal tool.


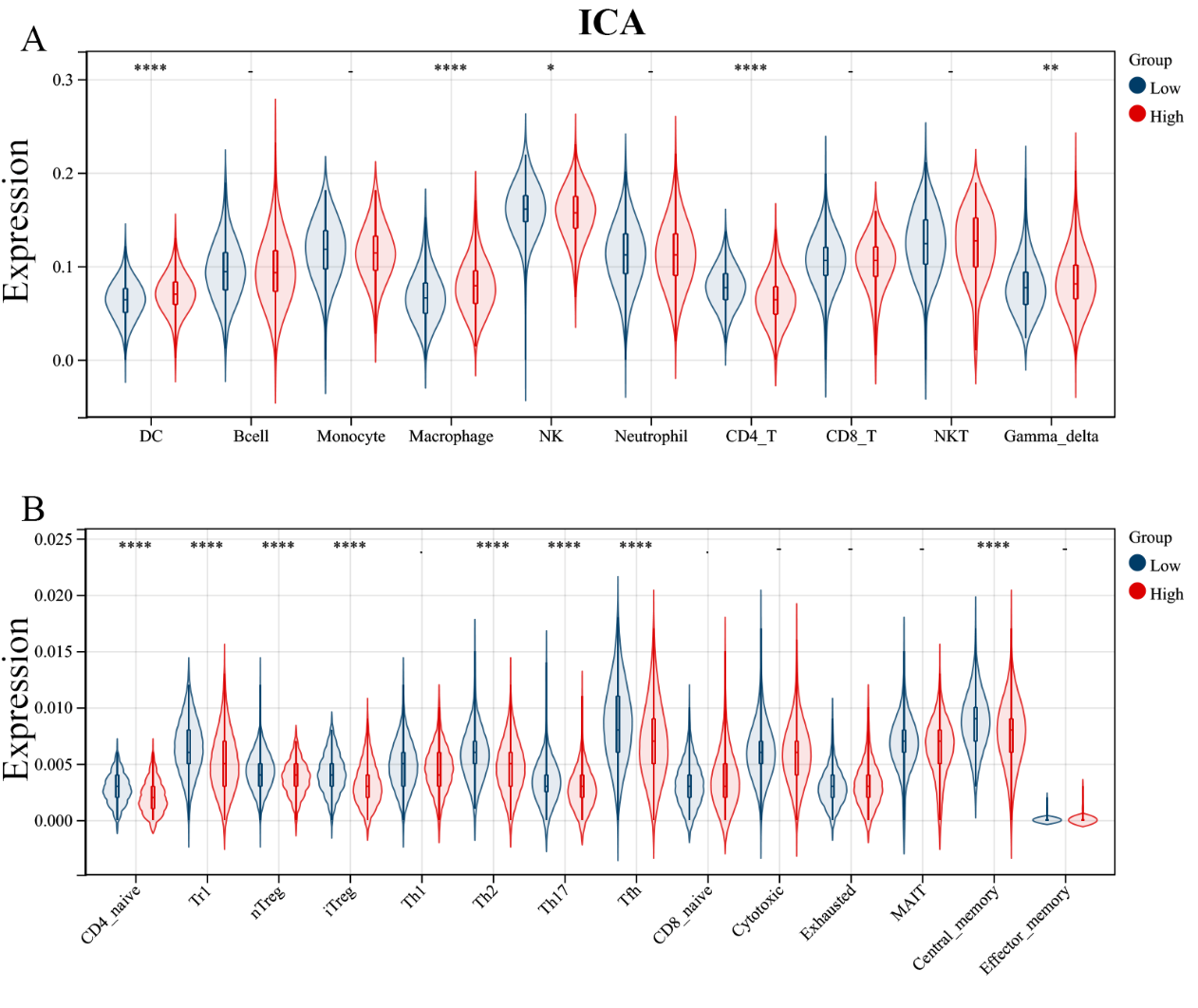


**Supplementary Figure 4. The correlations between CCNE1 expression and TIICs in UCEC via TCGA.**

**
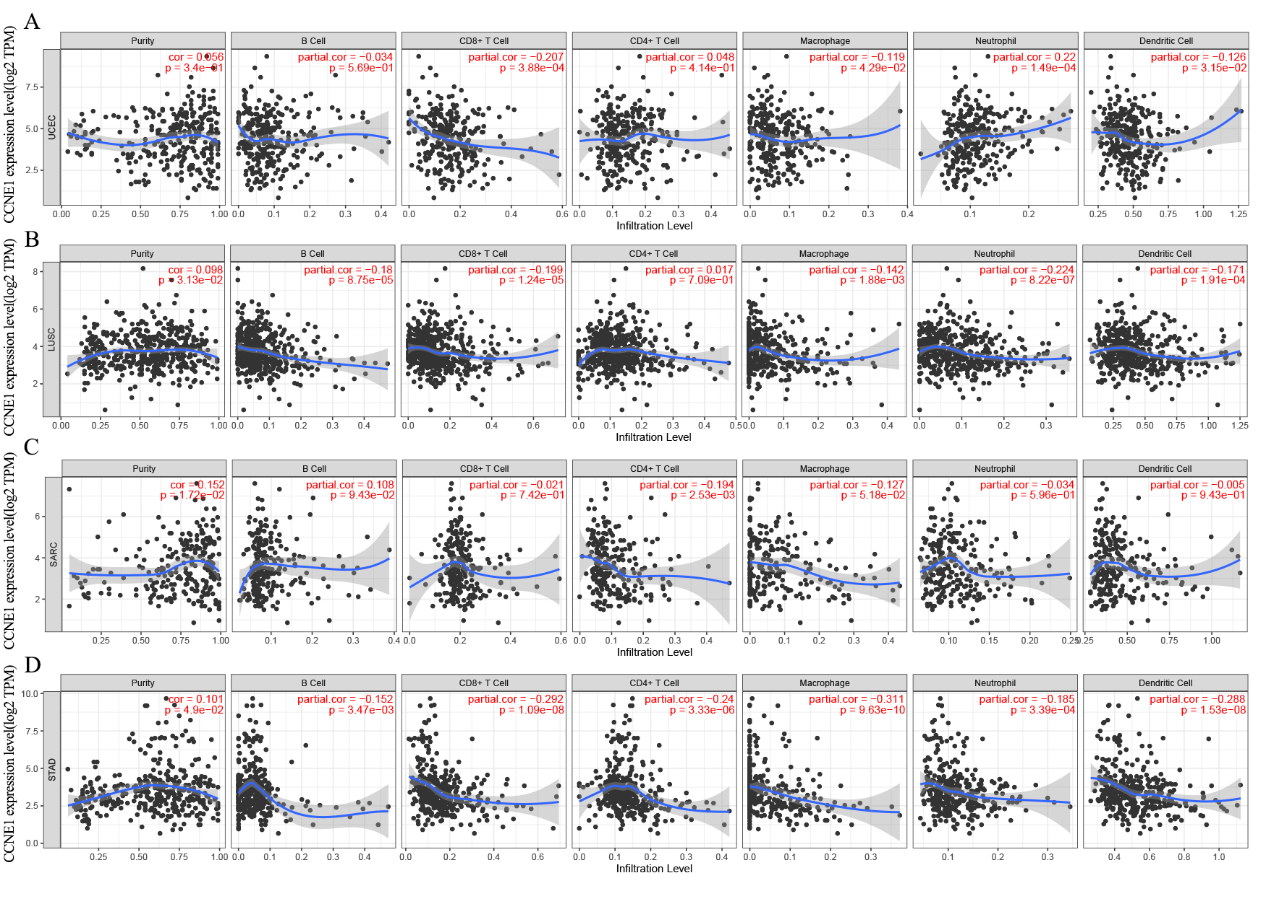
**

**Supplementary Figure 5. The correlations between CCNE1 expression and TIICs in UCEC (A), LUSC (B), SARC (C) and STAD (D) via TIMER portal.**
